# Downregulation of eukaryotic translation initiation factor 3b inhibited proliferation and metastasis of gastric cancer

**DOI:** 10.1038/s41419-019-1846-0

**Published:** 2019-08-19

**Authors:** Fang Ma, Xue Li, Juchao Ren, Ruiting Guo, Yuwei Li, Jichang Liu, Yundong Sun, Zhifang Liu, Jihui Jia, Wenjuan Li

**Affiliations:** 1 Key Laboratory for Experimental Teratology of Chinese Ministry of Education, The Shandong Provincial Key Laboratory of Infection and Immunology, Department of Microbiology, School of basic medical sciences, Shandong University-Karolinska Institutet Collaborative Laboratory for Cancer Research, Shandong University, Jinan, PR China; 2 Department of Urology, Qilu Hospital, Shandong University, Jinan, PR China; 3 Department of Biochemistry and Molecular Biology, School of basic medical sciences, Shandong University, Jinan, PR China

**Keywords:** Cancer, Oncogenesis

## Abstract

Eukaryotic translation initiation factor 3 (eIF3) plays an important role in the regulation of mRNA translation, cell growth and cancer development. eIF3b is the main scaffolding subunit in the eIF3 complex and has been demonstrated to contribute to the development of several cancers. First, our study found that the downregulation of eIF3b could inhibit the proliferation and metastasis of gastric cancer cells by regulating the expression of cancer-related genes. In addition, the expression of eIF3b correlated with the stage and progression of gastric cancer and was shown to be upregulated in human chronic gastritis and in gastric cancer tissues compared with the expression of eIF3b in normal gastric tissues. Moreover, *Helicobacter pylori* (*H. pylori*) infection could upregulate the expression of eIF3b in gastric cancer cells, suggesting that eIF3b might be involved in the carcinogenic process of *H. pylori*. The above findings identified the oncogenic role of eIF3b in gastric cancer development, and this may contribute to the exploration and discovery of novel therapeutic targets for gastric cancer treatment.

## Introduction

Gastric cancer is the fourth most frequently diagnosed cancer and ranks the third in cancer-related deaths worldwide^1^. Due to lack of reliable early diagnostic markers, most patients with gastric cancer are diagnosed at the advanced or metastatic stage^2^. Surgery, chemotherapy or radiotherapy have played only a minor role in improving the survival rate^3^. Therefore, it is urgent to identify new early diagnostic markers and to explore novel therapeutic targets^4^.

The occurrence and development of gastric cancer is a complicated multistep process^5^, and the regulation of genes mainly occurs at the level of transcription and translation^6^. At present, the study of protein translation, especially the regulation of the initial translation step, has demonstrated that eukaryotic initiation factors (eIFs) play a key role in protein translation, cell cycle regulation and the development of tumours^7^.

eIF3 is an important multisubunit complex in eIFs and consists of 13 subunits (eIF-3a, -3b, -3c…-3m)^8^. eIF3 binds to the 40S ribosomal subunit through interactions with other translation initiation factors, which can promote the formation of 43S initiation complex precursors, further bind to mRNA, recognize the initiation codon AUG, and initiate protein translation^9^. These functions are mainly based on six subunits of eIF3, namely eIF3a (p150), eIF3b, eIF3e, eIF3f, eIF3h and eIF3i^10^. It has been demonstrated that these six subunits are abnormally expressed in many tumours, and the differential expression is closely related to the progression of cancer^11^. Moreover, several subunits have been shown to be involved in the development of gastric cancer. eIF3a (p150) is overexpressed in gastric cancer, and its expression is associated with the clinicopathological parameters of gastric cancer^12^. eIF3h potentiates the cell proliferation and inhibits the cell apoptosis of gastric cancer cells^13^. The high expression of eIF3d is associated with a poor prognosis for patients with gastric cancer^14^. eIF3f expression is significantly decreased in many human cancers, and the decreased expression of eIF3f is a significant factor for a poor prognosis for gastric cancer patients^15–17^.

eIF3b is considered to be the main scaffolding subunit in the eIF3 complex^18^. Human eIF3b is a protein with an RNA recognition motif (RRM, located at the N-terminus), and the RRM domain provides a specific site for eIF3b to interact with eIF3J^19^. Some studies have suggested that eIF3b is involved not only in protein translation but also in cell proliferation, invasion, migration and tumour development. eIF3b has been shown to be overexpressed in prostate and bladder cancers, and its overexpression is associated with cancer prognosis^20^. In addition, the silencing of eIF3b can inhibit the proliferation of colon cancer cells and glioblastoma cells^21^. Moreover, eIF3b is abnormally expressed and plays an important role in the invasion and migration of cancer cells in osteosarcoma, oesophageal squamous cell carcinoma and renal cell carcinoma^22–24^. However, the expression of eIF3b and its effect on the progression of gastric cancer have not yet been reported. Therefore, this study aimed to investigate the role of eIF3b in the development of gastric cancer.

## Materials and methods

### Cell culture

Five human gastric cancer cell lines (SGC7901, MGC803, BGC823, HGC27 and AGS) and the human gastric epithelial cell line (GES-1) were purchased from the Cell Resource Center at the Shanghai Institute of Biochemistry and Cell Biology at the Chinese Academy of Sciences (Shanghai, China). SGC7901, MGC803, BGC823, HGC27 and GES-1 cells were cultured in RPMI-1640 medium plus 10% (v/v) foetal bovine serum (FBS). AGS cells were cultured in F12 medium plus 10% (v/v) FBS. The medium and FBS were purchased from Gibco/Life Technologies (Grand Island, NY, USA). The cell lines were all incubated in a humidified atmosphere with 5% CO_2_ at 37 °C.

### Small interfering RNA (siRNA) and transfection

The eIF3b siRNA and negative control siRNA were purchased from Suzhou Ribo Life Science Co., Ltd (Suzhou, China), which were transfected into SGC7901 and MGC803 cells, with Opti-MEM (Gibco/Life Technologies, Grand Island, NY, USA) as a transfection medium when the cell confluence was 40–50%. Lipofectamine 2000 (Invitrogen, Waltham, MA, USA) was used to transfect the siRNAs into the cells. The eIF3b and negative control siRNA sequences were as follows:

si-eIF3b-1 (Esi-1): 5′-GTGGGATATTCCAGAGAAA-3′.

si-eIF3b-2 (Esi-2): 5′-GGAGACTACTTGTGTGTGA-3′.

si-negative control (Csi): 5′-UUCUUCGAAACGUGUCACGUT-3′.

### Lentiviral-mediated shRNA vector and infection

The lentiviral-mediated eIF3b shRNA vector and the negative control shRNA vector were purchased from GeneChem (Shanghai, China) and were transfected into SGC7901 and MGC803 cells, respectively, when the cell confluence was 30–40% using Polybrene (GeneChem, Shanghai, China) as the infection reagent. The eIF3b and negative control shRNA sequence was as follows:

L.v-eIF3b: 5′-GACGTGAGCGAGGAAGAATTA-3′.

L.v-NC: 5′-TTCTCCGAACGTGTCACGT-3′.

### Plasmid transfection

The pENTER-E2F1 plasmid (GenePharma, Suzhou, China) was transfected into SGC7901 and MGC803 cells when the cell confluence was 80%. The pcDNA3.1-CagA plasmid was transfected into AGS cells, and the plasmid was kindly provided by Yongliang Zhu (Zhejiang University, China). The two plasmids were transfected with Polyplus Transfection jetPRIME Kit (Illkirch, France) according to the protocol.

### CCK-8 assay

The siRNA transfected cells were counted, and 1000 cells per well were plated in 96-well plates; there were three replicates for every treatment. At 24, 48, 72, 96 and 120 h after the transfection, 10 µl CCK-8 reagent (Dojindo Molecular Technologies, Japan) was added to the cell medium, and the samples were incubated for 3 h in the cell culture incubator; the absorbance at 450 nm was detected with a microplate reader (Eppendorf, Germany). The values obtained in every detection were used to generate cell proliferation curves.

### Colony formation assay

The siRNA transfected cells were counted, and 500 cells per well were plated in a new six-well plate. After 14 days of culture, the cell colonies were fixed with methanol for 30 min and were subsequently stained with a 0.5% crystal violet staining solution for 30 min. Finally, the cell colonies were photographed and counted to compare the proliferation abilities of the cells in the control and interference groups.

### Transwell assay

Matrigel (BD Biosciences, USA) was added to the transwell chambers, and the 24-well plate containing the chambers was placed into the cell culture incubator to speed up the solidification of Matrigel. After 4 h, the siRNA-transfected cells were counted, and 1 × 10^5^ cells per well were added into the chambers. We added 600 µl of medium plus 20% (v/v) FBS into the 24-well plate under the chamber, and the cells were suspended in serum-free medium inside of the chamber. After 24 h of incubation, the cells and Matrigel in the chambers were wiped off, and the cells on the other side of the chamber were fixed and stained. Finally, the cells were photographed and counted to compare the invasion of cells in the control and interference groups. When we detected the migration of the cells, no Matrigel was added, and the other steps were the same as above.

### Wound-healing assay

The cells were uniformly plated in 6-well plates, and 48 h after transfection, a 10 µl pipette tip was used to scratch the bottom of the plate by means of a ruler when the cells were evenly and densely spread. Then, the cell culture medium was changed to serum-free medium. After scratching, pictures of cell migration at different time points (0, 24 and 36 h) were taken under a microscope. Then, we calculated the percentage of scratching area in the total area and analysed the migration ability of the cells.

### Animal experiments

Four-week-old female BALB/c nude mice were purchased from Beijing Vital River Laboratory Animal Technology (Beijing, China). We randomly divided the nude mice into two groups, which were used for a subcutaneous injection (*n* = 7) and a tail vein injection (*n* = 16). SGC7901 cells were transfected with the lentiviral-mediated eIF3b shRNA and the negative control shRNA according to Multiplicity of Infection (MOI) 20:1 for 4 days. Then, we subcutaneously injected 1 × 10^6^ cells resuspended in 100 µl phosphate-buffered saline (PBS) into both sides of the backs of the nude mice. After 9 days, we measured the longest and shortest diameters of tumours every 2 days with callipers, which were, respectively, recorded as L and W, and the tumour volumes were calculated as *V* = *L* × *W*^2^ × 0.5. For the tail vein injection assay, ∼2 × 10^6^ SGC7901 cells resuspended in 100 µl PBS were injected into the tail veins of nude mice. We observed the metastasis of cells in nude mice using a small-animal in vivo imaging system.

### Specimens

This study collected 39 specimens of chronic gastritis tissues and normal gastric mucosal tissues and 43 pairs of gastric cancer tissues and non-tumorous adjacent tissues. Some tissues were stored in RNAlater solution, placed in a refrigerator at 4 °C overnight and transferred into a freezer at −80 °C for storage. The remaining tissues were stored in formalin for immunohistochemistry analysis. All tissues from patients were obtained from Qilu Hospital at Shandong University (Jinan, China). This study was approved by the Ethics Committee of Shandong University.

### *Helicobacter pylori* culture and cell infection

*H. pylori* *11637* (*Hp11637*) and *H. pylori* *26695* (*Hp26695*) were kindly provided by Dr Jianzhong Zhang (Chinese Disease Control and Prevention Center, China). The two *H. pylori* strains were cultivated on Brucella agar plates containing 5% (v/v) Defibrinated Sheep Blood at 37 °C under microaerobic conditions (5% O_2_, 10% CO_2_ and 85% N_2_). *H. pylori* were collected and resuspended in PBS. The quantity of bacteria was analysed by spectrophotometry. AGS cells were infected with *Hp11637* and *Hp26695* for 0, 6, 12 and 24 h at an MOI of 100:1. Then, two *H. pylori* strains were used to infect AGS cells for 8 h at MOIs of 0, 50:1, 100:1 or 150:1.

### RNA preparation, reverse transcription PCR and qRT-PCR

TRIzol Reagent (Invitrogen, Waltham, MA, USA) was used to extract the total RNA from cells and tissues, and the concentration and purity of the total RNA were detected by an ultraviolet spectrophotometer (Eppendorf, Germany). The RNA was reverse transcribed into cDNA with PrimeScript^TM^ RT Reagent Kit with gDNA Eraser (Perfect Real Time) (Takara, Japan). Real-time PCR was conducted using SYBR Premix Ex Taq System (Takara), using LightCycler® 2.0 Real-time PCR System (Roche, USA). The results were determined using the 2^−ΔΔCt^ method. The primer sequences of eIF3b were as follows: 5′-CGGTGCCTTAGCGTTTGTG-3′ (forward) and 5′-CGGTCCTTGTTGTTCTTCTGC-3′ (reverse); the primer sequences of GAPDH were 5′-TGACTTCAACAGCGACACCCA-3′ (forward) and 5′-CACCCTGTTGCTGTAGCCAAA -3′ (reverse).

### Western blot analysis

The cells were collected and lysed with RIPA lysis buffer with the proteinase inhibitor PMSF (Solarbio, China) at a ratio of 100:1 (v/v). The protein concentration was determined by the BCA reagent kit (Beyotime, China). Equal amounts of protein were separated by 10% SDS-PAGE and were transferred to PVDF membranes, which were incubated with antibodies against eIF3b (Abcam, USA) and β-actin (Cell Signaling Technology, USA) at 4 °C overnight. An anti-mouse horseradish peroxidase antibody was used as a secondary antibody, and the membranes were incubated at room temperature for an hour. Finally, the PVDF membranes were developed with the enhanced chemiluminescence method (ECL, Millipore) and detected by a chemiluminometer (Bio-Rad, USA).

### ELISA

The cell supernatants were collected after transfection and were centrifuged at 1000 rpm for 4 min at 4 °C. Then, the supernatants were transferred to a new EP tube for use. The IL-8 protein expression level was detected according to the instructions of the Human IL-8 ELISA Kit (Neobioscience, China). Finally, the absorbance of IL-8 was detected by spectrophotometry at 450 nm, and the resulting values were expressed in pg/ml.

### Immunohistochemistry

Formalin-fixed tissues were embedded into paraffin and were sectioned by the Department of Pathology of Qilu Hospital (Jinan, China). First, the tissue sections were deparaffinized, and antigen retrieval was performed. Then, the sections were incubated with a primary antibody against eIF3b (Abcam, USA) at 4 °C. The next day, the sections were incubated with an anti-mouse secondary antibody and were developed with the DAB Kit (Gene Tech, Shanghai, China) according to the instructions.

### Statistical analysis

Statistical analyses were performed with GraphPad Prism and SPSS. Comparisons between the different groups were analysed by Student’s *t*-test. The relationship between eIF3b mRNA expression in specimen tissues and the clinicopathological parameters were analysed using the *χ*^2^ test. *P* < 0.05 was considered statistically significant.

## Results

### The eIF3b-specific siRNA significantly decreased the expression of eIF3b in gastric cancer cells

**Fig. 1:**
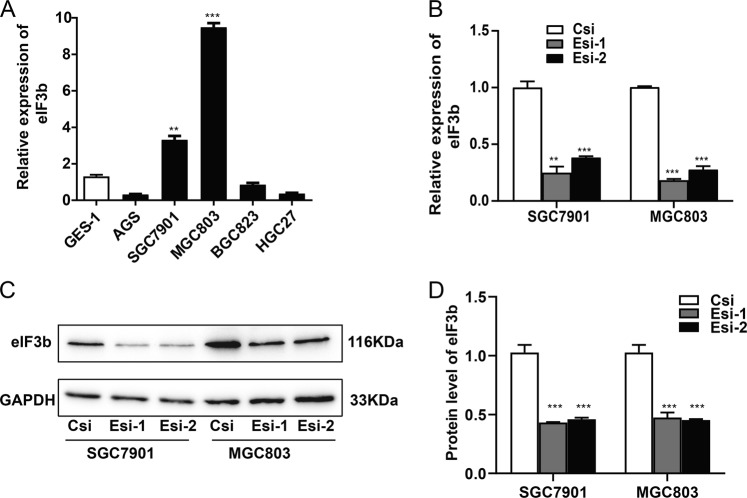
The eIF3b-specific siRNA significantly decreased the expression of eIF3b in SGC7901 and MGC803 cells. **a** Compared with that of the human immortalized gastric epithelial cell line GES-1, the expression of eIF3b mRNA was higher in the gastric cancer cell lines SGC7901 and MGC803. **b** qRT-PCR analysis showed that the eIF3b siRNAs significantly decreased the mRNA expression of eIF3b in SGC7901 and MGC803 cells. **c** Western blot analysis showed that the eIF3b siRNAs significantly decreased the protein expression of eIF3b in SGC7901 and MGC803 cells compared with that in the control cells. This experiment was repeated three times. **d** The grey scale of the protein bands was analysed with ImageJ software. The data are the means ± SDs of three independent experiments. The differences were statistically significant. **P < 0.01, ***P < 0.001

qRT-PCR was used to detect the expression of eIF3b mRNA in the human immortalized gastric epithelial cell line GES-1 and in five other gastric cancer cell lines, AGS, SGC7901, MGC803, BGC823 and HGC27. The results show that the mRNA expression of eIF3b was higher in the gastric cancer cell lines SGC7901 and MGC803 compared with the mRNA expression in the other cell lines. Therefore, we chose the SGC7901 and MGC803 cell lines for the following cell function experiments (Fig. 1a).

The two different eIF3b siRNAs were transiently transfected into SGC7901 and MGC803 cells. After transfection for 48 h, qRT-PCR analysis shows that the two eIF3b siRNAs could both significantly decrease the mRNA expression of eIF3b in SGC7901 and MGC803 cells (Fig. 1b). After transfection for 72 h, western blot (WB) analysis shows that the two eIF3b siRNAs could also decrease the protein expression of eIF3b in SGC7901 and MGC803 cells (Fig. 1c, d). This experiment was repeated three times, and the grey scale of the protein bands was analysed with ImageJ software. The difference was statistically significant.

### Downregulation of eIF3b inhibited the proliferation, invasion and migration of gastric cancer cells in vitro

**Fig. 2:**
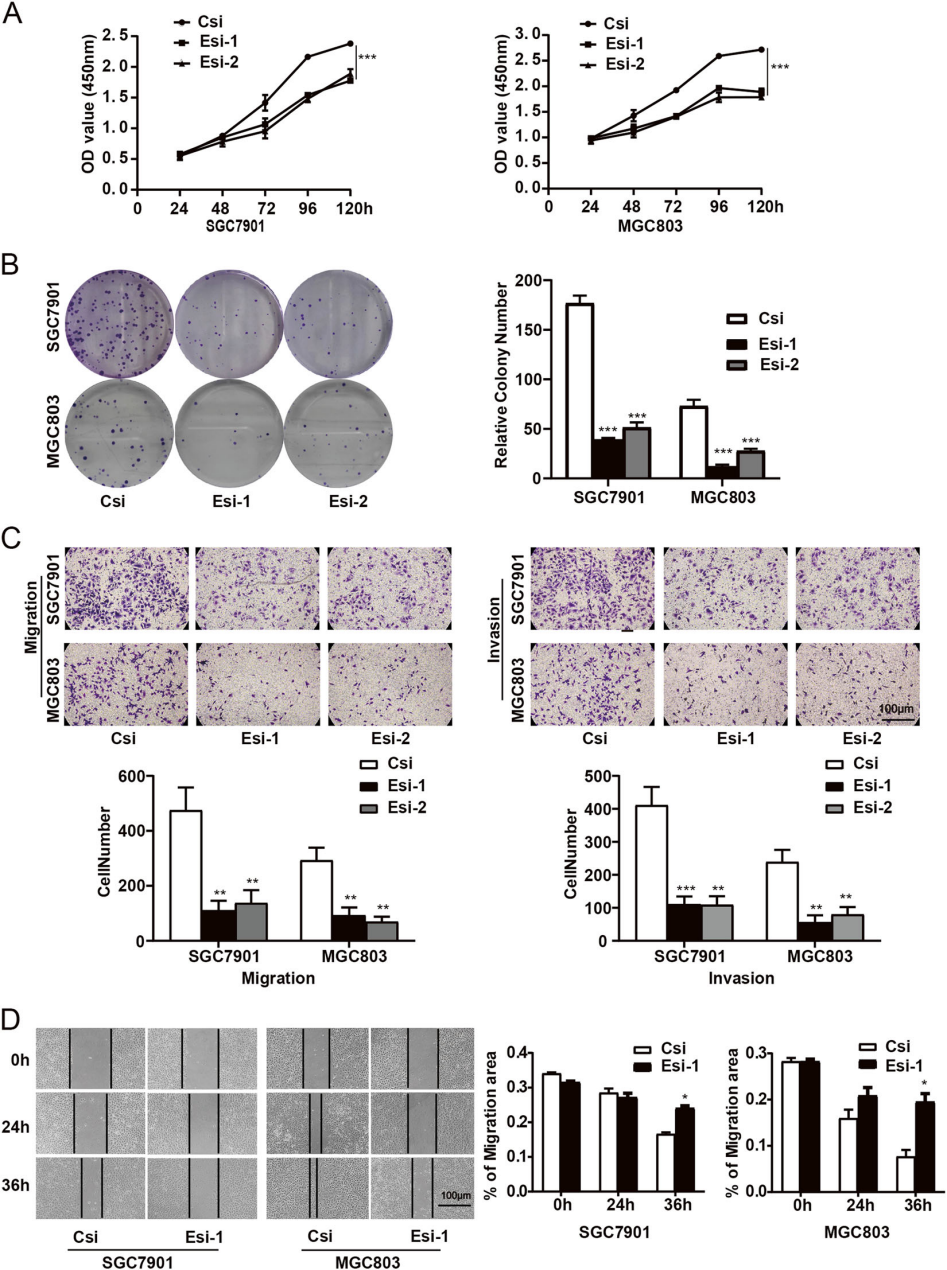
Downregulation of eIF3b inhibited the cell proliferation, invasion and migration abilities of gastric cancer cells in vitro. **a** A CCK-8 assay measured the OD values at 5 time points (24, 48, 72, 96 and 120 h). The analysis showed that the downregulation of eIF3b inhibited the proliferation of SGC7901 and MGC803 cells. **b** A colony-formation assay showed that the downregulation of eIF3b inhibited the clonogenic abilities of SGC7901 and MGC803 cells. **c** Transwell assays showed that the downregulation of eIF3b inhibited the migration and invasion abilities of SGC7901 and MGC803 cells. **d** The percentage of the scratching area in the total area (% of migration area) was analysed in a wound-healing assay, and the results showed that the downregulation of eIF3b inhibited the migration of SGC7901 and MGC803 cells. The data are the means ± SDs of three independent experiments. *P < 0.05, **P < 0.01, ***P < 0.001

CCK-8 and colony-formation assays were used to verify the effects of the downregulation of eIF3b on the proliferation of gastric cancer cells. We found that, compared with those of the control groups, SGC7901 and MGC803 cells with eIF3b inhibition showed low proliferative abilities, indicating that the downregulation of eIF3b inhibited the proliferation of gastric cancer cells (Fig. 2a, b).

A transwell assay was used to detect the effect of eIF3b inhibition on the migration and invasion of gastric cancer cells. The results show that the number of SGC7901 and MGC803 cells migrating and invading to the other side of the membranes was significantly reduced in the experimental groups compared with the number of cells migrating and invading in the control groups; this indicates that the downregulation of eIF3b inhibited the invasion and migration of gastric cancer cells (Fig. 2c).

A wound-healing assay was used to detect the effect of downregulating eIF3b expression on the migration of gastric cancer cells. The results show that in the experimental groups, the number of SGC7901 and MGC803 cells migrating to the scratches was lower than that of the control groups; in addition, the percentages of the scratching area in the total area in the experimental groups were larger than those in the control groups, also indicating that the downregulation of eIF3b inhibited the migration of gastric cancer cells (Fig. 2d).

### Downregulation of eIF3b inhibited the proliferation and metastasis of gastric cancer cells in vivo

**Fig. 3:**
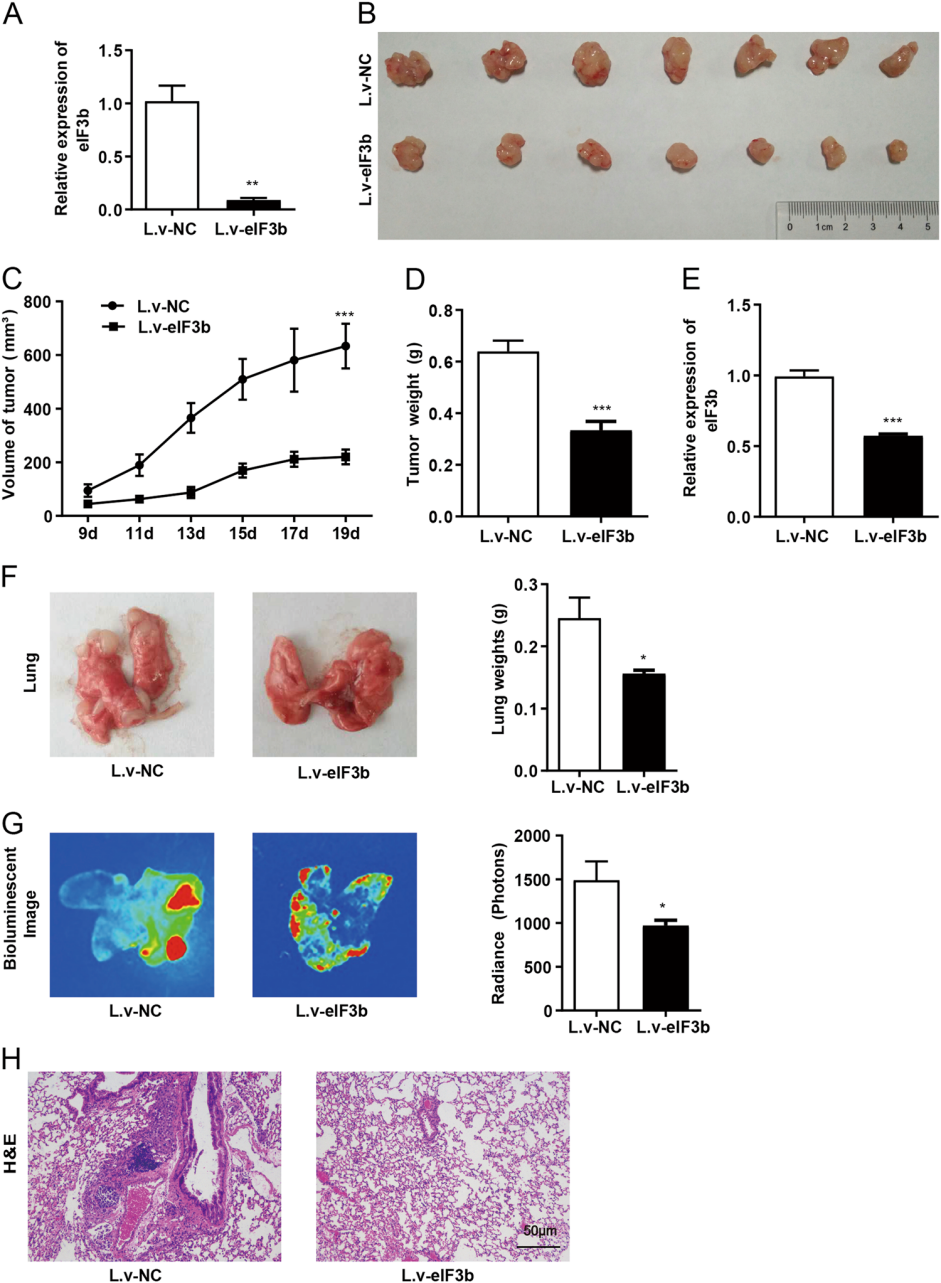
Downregulation of eIF3b inhibited the proliferation and metastasis of gastric cancer cells in vivo. Xenograft assay in nude mice: **a** qRT-PCR results showed that the mRNA expression of eIF3b in the cells infected with eIF3b interfering lentivirus (L.v-eIF3b) was inhibited. **b**, **c** Starting from the 9th day, the volume of the tumour blocks was measured every other day, and the nude mice were sacrificed after 19 days. The tumours grew more slowly, and the tumour blocks were smaller in the eIF3b inhibition group compared with those in the control group. **d** The analysis of the tumour weight showed that the downregulation of eIF3b inhibited the proliferation of SGC7901 cells in nude mice. **e** The qRT-PCR results showed that the mRNA expression level of eIF3b in the tumours in the experimental groups (L.v-eIF3b) was inhibited. Tail vein injection experiment in nude mice: **f** After 2 months, the nude mice were sacrificed, and the tumours in the lungs were fewer and smaller in the eIF3b inhibition group mice compared with the number and size of the tumours in the control group mice. **g** The bioluminescent images and fluorescence intensities of the lung tissues showed that the downregulation of eIF3b inhibited the metastasis of SGC7901 cells. **h** H&E staining of the lung tissues showed that the infiltration and metastasis of cancer cells were more obvious in the control groups (L.v-NC) than they were in the experimental groups (L.v-eIF3b). *P < 0.05, **P < 0.01, ***P < 0.001

SGC7901 cells were infected with an RNA-interfering lentivirus vector and qRT-PCR was used to detect the expression of eIF3b in the lentivirus infected cells (Fig. 3a). Then, the cells were injected subcutaneously into nude mice. After 7 days, tiny tumours appeared, and from the 9th day, the volume of tumour was measured every other day. The tumour growth curve shows that the downregulation of eIF3b inhibited the growth of subcutaneous xenografts in nude mice (Fig. 3b, c). After 19 days, the nude mice were sacrificed, and the weights of the tumour tissues in the experimental groups were significantly less than those in the control groups (Fig. 3d). qRT-PCR was used to detect the expression of eIF3b in the tumour tissues, and the results show that the eIF3b mRNA expression in the experimental groups was inhibited (Fig. 3e). The subcutaneous injection test indicated that the downregulation of eIF3b inhibited the proliferation of gastric cancer cells in vivo.

SGC7901 cells were transfected with an RNA-interfering lentivirus vector and were injected into the tail veins of nude mice. After 2 months, the SGC7901 cells that were labelled with GFP transferred to the lung, and then the nude mice were sacrificed. In the experimental groups, the tumour nodules were fewer and smaller, the weights of the lung tissues were lighter and the fluorescence intensity of lung tissues was weaker than those in the control groups (Fig. 3f, g). Then, the paraffin-embedded lung tissues were sectioned and stained, and the H&E staining results show that in the control group, the infiltration and metastasis of cancer cells in the lung tissues were more obvious than those in the experimental group (Fig. 3h). From those analyses, we concluded that the downregulation of eIF3b inhibited the metastasis of gastric cancer cells in vivo.

### eIF3b regulated the protein expression of key genes in the signalling pathways related to gastric cancer

**Fig. 4:**
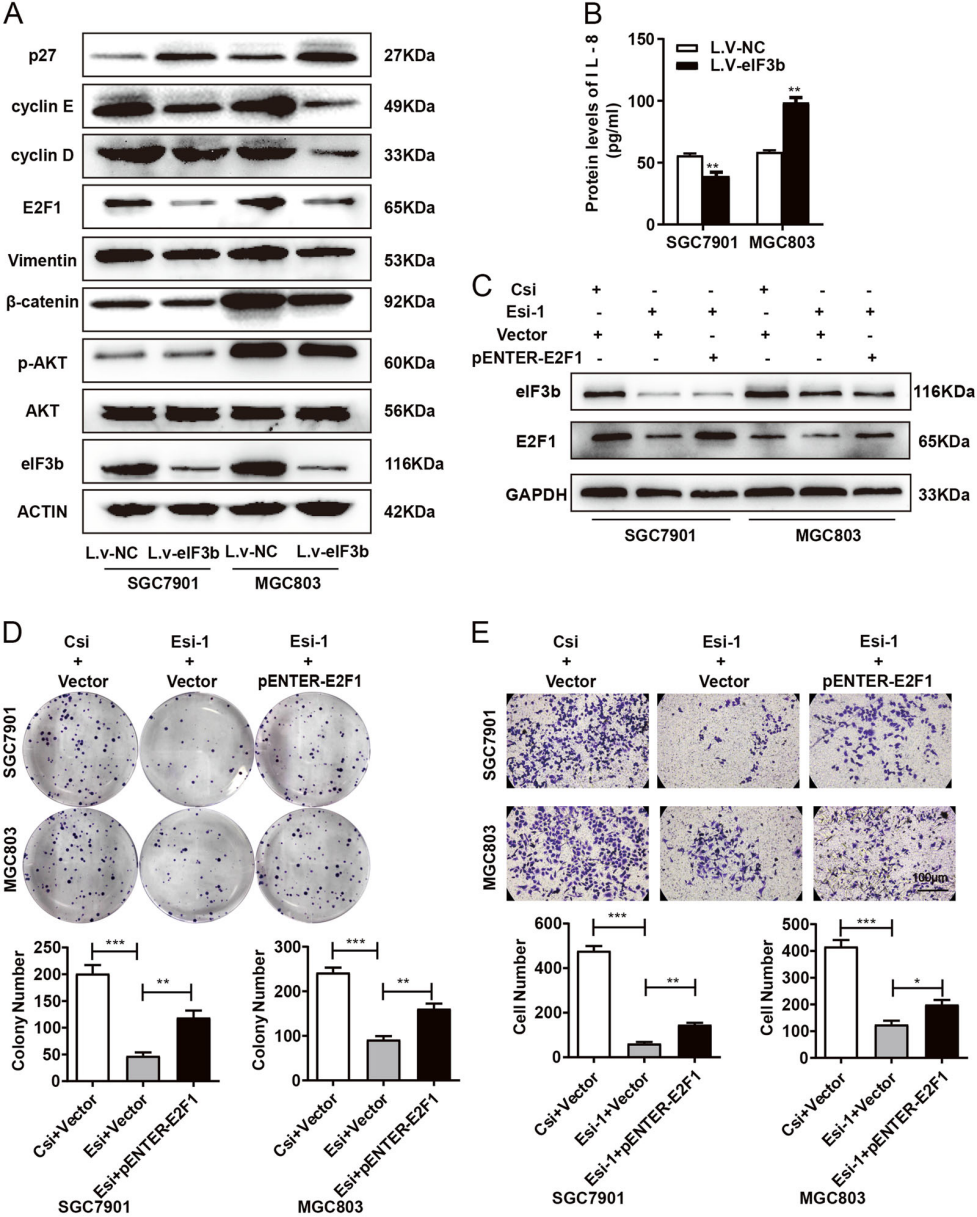
eIF3b regulated the protein expression of key genes in the cancer pathway. **a** The western blot results showed that the downregulation of eIF3b inhibited the expression of E2F1, cyclin D, cyclin E, vimentin and β-catenin and increased the expression of the tumour suppressor gene P27 in SGC7901 and MGC803 cells. **b** The ELISA results showed that the downregulation of eIF3b inhibited the expression of IL-8 in SGC7901 cells but increased the expression of IL-8 in MGC803 cells. **c** The WB results showed that the pENTER-E2F1 plasmid increased the expression of E2F1. **d**, **e** The colony formation and transwell assays showed that the role of eIF3b in the proliferation and migration of SGC7901 and MGC803 cells was partially restored by E2F1. *P < 0.05, **P < 0.01, ***P < 0.001

As an important translation initiation factor, eIF3b plays an important role in protein translation. The WB results show that the downregulation of eIF3b inhibited the expression of E2F1, cyclin E, cyclin D, vimentin and β-catenin and increased the expression of the tumour suppressor gene P27 in SGC7901 and MGC803 cells (Fig. 4a). The ELISA results show that downregulating the expression of eIF3b inhibited the expression of IL-8 in SGC7901 cells and increased the expression of IL-8 in MGC803 cells (Fig. 4b). These protein molecules are directly or indirectly involved in the proliferation and metastasis of gastric cancer cells. Because the regulation of IL-8 expression was inconsistent in the two cell lines, we speculated that it was related to the different backgrounds of the cell lines. To further verify that the downregulation of eIF3b indeed regulated the proliferation and metastasis of gastric cancer cells by affecting the expression levels of key genes, we selected E2F1, whose expression was dramatically decreased by eIF3b inhibition, to perform the rescue experiments. We then constructed a pENTER-E2F1 plasmid, which was cotransfected with siRNA-eIF3b into SGC7901 and MGC803 cells. The WB results show that the pENTER-E2F1 plasmid could increase the expression of E2F1 (Fig. 4c). Colony formation and transwell assays show that the increase in E2F1 expression could partially restore the proliferation and migration of SGC7901 and MGC803 cells with eIF3b inhibition; this indicates that the role of eIF3b in the proliferation and migration of gastric cancer cells was partially dependent on E2F1 (Fig. 4d, e).

### Overexpression of eIF3b in human chronic gastritis and gastric cancer specimens

**Fig. 5:**
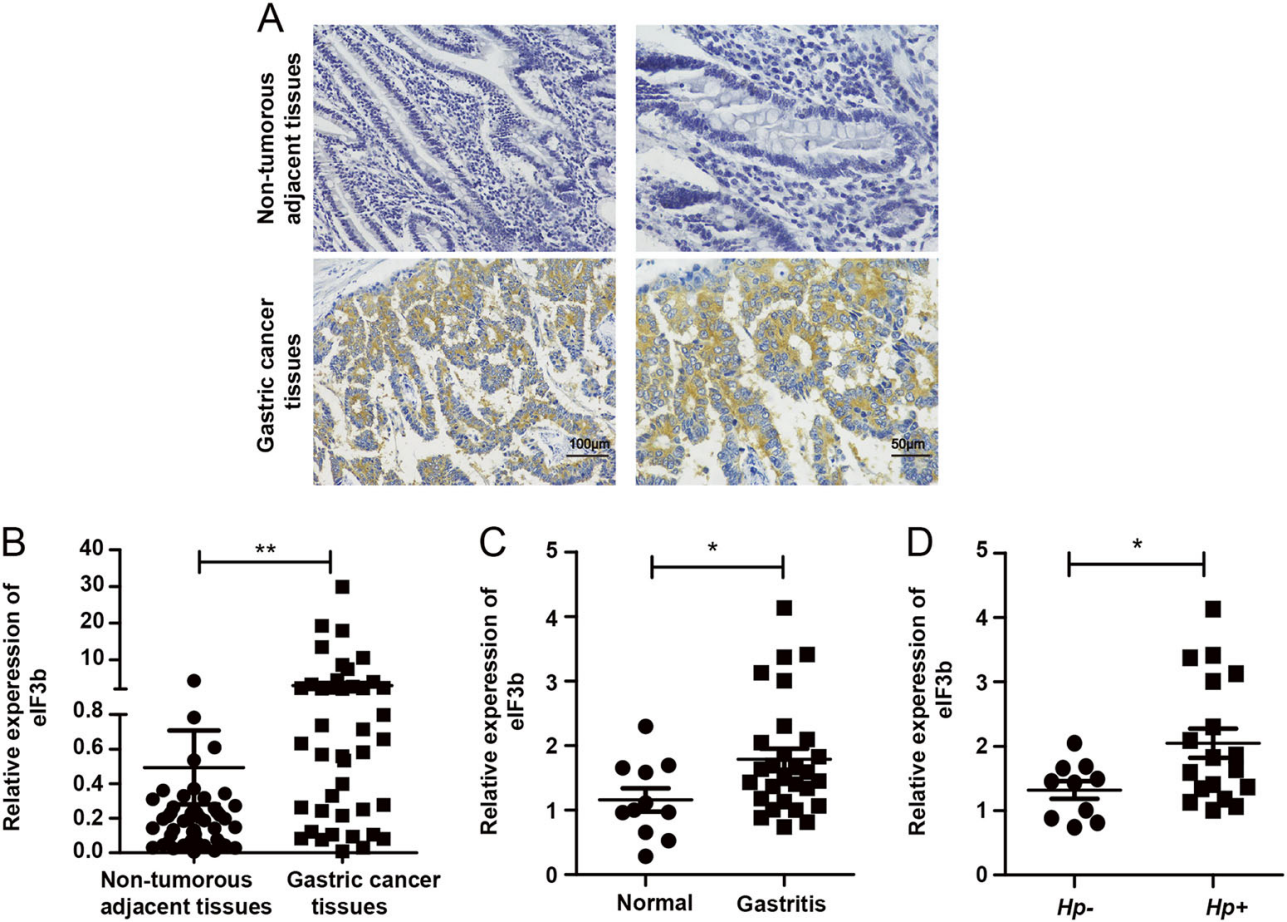
Overexpression of eIF3b in human chronic gastritis and gastric cancer specimens. **a** The immunohistochemistry results showed that the eIF3b protein was highly expressed in the gastric cancer tissues and that its expression was mainly distributed in the cytoplasm. **b** The qRT-PCR results showed that the eIF3b mRNA was overexpressed in the gastric cancer tissues. **c**, **d** The qRT-PCR results showed that the eIF3b mRNA was highly expressed in chronic gastritis tissues compared with the expression in normal gastric tissues and that the eIF3b mRNA was higher in the *Hp*+ gastritis tissues than it was in the *Hp*− gastritis tissues. *P < 0.05, **P < 0.01

To verify the correlation between the aforementioned experimental results and clinical findings, we used immunohistochemistry and qRT-PCR to detect the protein and mRNA expression of eIF3b, respectively. The immunohistochemistry results show that the eIF3b protein was overexpressed in gastric cancer tissues, and its expression was mainly distributed in the cytoplasm (Fig. 5a). The qRT-PCR results show that the eIF3b mRNA levels in gastric cancer tissues were higher than those in non-tumorous adjacent tissues (Fig. 5b). In addition, we analysed the correlation between the mRNA expression of eIF3b and the clinicopathological parameters of patients with gastric cancer. The analysis shows that the mRNA expression of eIF3b was not related to the patient’s age or gender but was related to the stage and progression of the tumour (Table 1). In addition, the qRT-PCR results show that the eIF3b mRNA expression in chronic gastritis tissues was higher than that in normal gastric mucosal tissues (Fig. 5c). The chronic gastritis tissues were divided into *Hp*-positive (*Hp*+) and *Hp*-negative (*Hp*−) groups according to the results of gastroscopy. Then, we performed a statistical analysis again and found that the mRNA expression of eIF3b was higher in *Hp*+ gastritis tissues than that in *Hp*− gastritis tissues (Fig. 5d).

**Table 1:** Correlations between eIF3b expression and the clinicopathological parameters of GC patients

Clinicopathological features	Total	eIF3b mRNA expression		*P* value
	(pairs)	Low	High	
	43	13	30	
Age				
≥61	24	8	16	0.076
<61	19	5	14	
Gender				
Male	25	9	16	0.146
Female	18	4	14	
Histological grade				
I–II	16	5	11	0.017*
III–IV	27	8	19	
TNM stage				
I–II	9	3	6	0.035*
III–IV	34	10	24	

*P < 0.05

### *H. pylori* infection upregulated eIF3b expression in gastric cancer cells

**Fig. 6:**
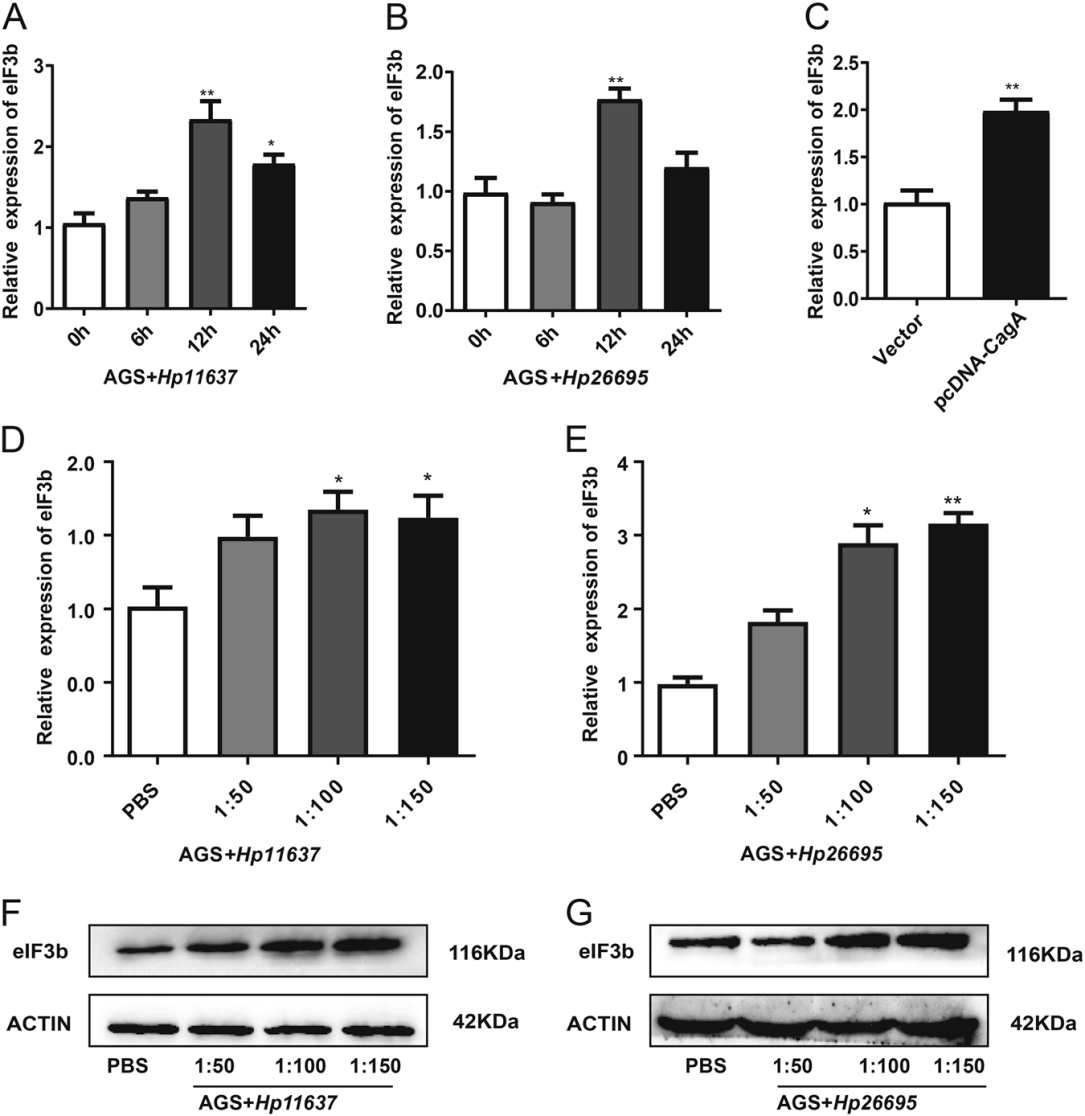
*H. pylori* infection upregulated eIF3b expression in gastric cancer cells. **a**, **b** The qRT-PCR results showed that the infections with the *Hp11637* and *Hp26695* strains both upregulated the mRNA expression of eIF3b in AGS cells at an MOI of 100:1, especially 12 h after the infection. **c** The qRT-PCR results showed that CagA could upregulate the mRNA expression of eIF3b in AGS cells. **d–g** The qRT-PCR and western blot results showed that the *Hp11637* and *Hp26695* strains could upregulate the mRNA and protein expression of eIF3b in AGS cells in a dose-dependent manner. The data are the means ± SDs of three independent experiments. *P < 0.05, **P < 0.01

AGS cells were infected with *Hp11637* and *Hp26695* at a ratio of 100:1, and the qRT-PCR results show that both of the *H. pylori* strains upregulated the expression of eIF3b mRNA in AGS cells, especially 12 h after the infection (Fig. 6a, b). Next, we verified whether CagA, an important virulence factor of *H. pylori*, can upregulate the expression of eIF3b in AGS cells. The pcDNA3.1-CagA plasmid was transfected into AGS cells. After 48 h, qRT-PCR detection shows that CagA could upregulate the expression of eIF3b mRNA in AGS cells (Fig. 6c). Then, we used *Hp11637* and *Hp26695* to infect AGS cells at the ratios of 50:1, 100:1 and 150:1 for 12 h. The qRT-PCR and WB results show that the eIF3b mRNA and protein levels were upregulated in AGS cells after *H. pylori* infection in a dose-dependent manner (Fig. 6d–g).

## Discussion

Several studies have shown that eIF3b can affect the development of cancer, but the role of eIF3b in gastric cancer is not clear. We first demonstrated that silencing eIF3b expression affected the proliferation, migration and invasion abilities of gastric cancer cells. Then, by constructing a xenograft model, it was confirmed that the inhibition of eIF3b could also inhibit tumour proliferation and metastasis in vivo.

In gastric cancer cells, after interference with the expression levels of eIF3b, E2F1, cyclin D and cyclin E was downregulated. According to our results, we hypothesized that eIF3b might affect cell proliferation partially by regulating E2F1. E2F1 is a member of the E2F family of transcription factors. The E2F family plays a key role in the regulation of the cell cycle and is also one target of the small DNA tumour virus transforming proteins. E2F1, as well as E2F2 and E2F3, has an additional cyclin binding domain. This domain preferentially binds to the retinoblastoma protein pRB in a cell cycle-dependent manner. It can also mediate cell proliferation and apoptosis in a p53-dependent or p53-independent manner^25^. The results of the rescue experiments also demonstrated that the role of eIF3b in proliferation is related to the E2F1 signalling pathway and may affect cell proliferation by p53-independent regulation.

IL-8 could be involved in the NF-κB signalling pathway to affect the proliferation of gastric cancer cells, and it also played an important role in the formation of tumour neovascularization^26,27^. From the analysis of these genes in gastric cancer-related signalling pathways, we found that the regulatory trend of the IL-8 protein due to eIF3b inhibition is inconsistent between the SGC7901 and MGC803 cells. We speculated that this might be related to the difference in the cell backgrounds, as SGC7901 is derived from a P53 mutant gastric cancer cell line^28^, and MGC803 is derived from a P53 wild-type gastric cancer cell line^29^. To verify our speculation, we downregulated eIF3b expression in AGS cells (a gastric cancer cell line with wild-type P53^30^). The expression of IL-8 was detected, and the results show that the downregulation of eIF3b in AGS cells also increased the expression of IL-8 (data not shown), which was consistent with the results in MGC803 cells. The results indicate that the regulation of IL-8 by eIF3b might be dependent on P53, but further studies should be performed to resolve this question. In oesophageal squamous cell carcinoma, eIF3b promoted cancer progression by activating the β-catenin signalling pathway^22^. We also found that eIF3b could affect the expression of β-catenin and vimentin in gastric cancer cells. We will further investigate whether eIF3b affects cell invasion and migration through EMT or the Wnt/β-catenin pathway in gastric cancer.

EIF3b is one of the subunits of eIF3. We were interested in analysing if the effect of eIF3b on gastric cancer cells depends on the translation functions of eIF3. EIF3b, a universal translation initiation factor subunit, is not specific to a certain class of target proteins, and it has been reported that the silencing of eIF3b causes a decrease in the total cellular protein level^20^. We found that eIF3b inhibition downregulated the expression of some types of protein but upregulated the expression of others in gastric cancer cells. Moreover, the inhibition of eIF3b expression can also regulate the mRNA expression level of some genes in gastric cancer (data not shown). Therefore, the regulation of eIF3b on gene expression could be in a direct or indirect way. It has been found that the expression levels of not all translation initiation factor subunits are upregulated in gastric cancer. For example, eIF3f is downregulated in various cancers, including gastric cancer^16,31,32^. Whether eIF3b cooperates with other eIF3 subunits to promote gastric cancer progression needs to be further studied.

Both cell line and animal studies have suggested a role for eIF3b in the development of gastric cancer. Whether the expression of eIF3b is clinically relevant is a matter of great concern to us. According to our results, compared with that in normal gastric tissues, the eIF3b mRNA expression was increased in gastritis tissues and gastric cancer tissues. Moreover, the expression of eIF3b in *Hp*+ gastritis tissues was higher than the expression in *Hp*− gastritis tissues. The immunohistochemistry results show that the expression of the eIF3b protein in gastric cancer tissues was significantly higher than that in non-tumorous adjacent tissues and that eIF3b was mainly expressed in the cytoplasm. Next, we analysed the correlation between the eIF3b mRNA expression level and the clinical pathological parameters and found that the expression of eIF3b is related to the progression and clinical stage of gastric cancer, suggesting that eIF3b may be a new diagnostic marker and therapeutic target for gastric cancer.

Nearly half of the world’s population has *H. pylori* colonization in the stomach^33^. *H. pylori* infection plays an important role in promoting the development of gastric inflammatory diseases, ulcers and gastric-related malignancies^34^. *H. pylori* infection is an important initiation factor for the malignant transformation of gastric mucosal tissues^35^. It has been shown that *H. pylori* infection can promote the proliferation and colony-forming abilities of gastric cancer cells and can induce the secretion of the inflammatory factor IL-8^36^. Thus, we detected eIF3b expression in gastric cancer cells in the presence of *H. pylori*. At different time points or different MOI values, the expression level of eIF3b was significantly higher in AGS cells cocultured with the *Hp26695* and *Hp11637* strains than that in the control group. The CagA virulence factor of *H. pylori* could also upregulate the expression of eIF3b in AGS cells. Due to the limitations of the in vitro experiments, we may need to validate the effect of *H. pylori* on the expression of eIF3b in animal experiments. The above results indicate that eIF3b might be involved in the pathogenesis of *H. pylori*.

In summary, our study reveals the role of eIF3b in the development of gastric cancer at both the in vivo and in vitro levels. In addition, we also examined the expression of eIF3b and its correlation with clinicopathological parameters in clinical tissue samples; this provides a basis for evaluating eIF3b as a potential diagnostic or prognostic marker for gastric cancer. However, more studies are needed to address the specific regulatory mechanism of eIF3b in gastric cancer.

## References

[1] Bray F (2018). Global cancer statistics 2018: GLOBOCAN estimates of incidence and mortality worldwide for 36 cancers in 185 countries. CA Cancer J. Clin..

[2] Sharma MR, Schilsky RL (2011). GI cancers in 2010: new standards and a predictive biomarker for adjuvant therapy. Nat. Rev. Clin. Oncol..

[3] Wu CW (2000). Surgical mortality, survival, and quality of life after resection for gastric cancer in the elderly. World J. Surg..

[4] Nam DH (2013). Prognostic value of early postoperative tumor marker response in gastric cancer. Ann. Surg. Oncol..

[5] D’Elia L, Rossi G, Ippolito R, Cappuccio FP, Strazzullo P (2012). Habitual salt intake and risk of gastric cancer: a meta-analysis of prospective studies. Clin. Nutr..

[6] Mihmanli M, Ilhan E, Idiz UO, Alemdar A, Demir U (2016). Recent developments and innovations in gastric cancer. World J. Gastroenterol..

[7] Holcik M, Sonenberg N (2005). Translational control in stress and apoptosis. Nat. Rev. Mol. Cell Biol..

[8] Lee AS, Kranzusch PJ, Cate JH (2015). eIF3 targets cell-proliferation messenger RNAs for translational activation or repression. Nature.

[9] Simonetti A (2016). eIF3 peripheral subunits rearrangement after mRNA binding and start-codon recognition. Mol. Cell..

[10] Hinnebusch AG (2006). eIF3: a versatile scaffold for translation initiation complexes. Trends Biochem Sci..

[11] Valášek LS (2017). Embraced by eIF3: structural and functional insights into the roles of eIF3 across the translation cycle. Nucleic Acids Res..

[12] Chen G, Burger MM (2004). p150 overexpression in gastric carcinoma: the association with p53, apoptosis and cell proliferation. Int J. Cancer.

[13] Wang X (2018). Eukaryotic translation initiation factor EIF3H potentiates gastric carcinoma cell proliferation. Tissue Cell..

[14] He J, Wang X, Cai J, Wang W, Qin X (2017). High expression of eIF3d is associated with poor prognosis in patients with gastric cancer. Cancer Manag. Res..

[15] Cheng Y, Zhou J, Li H (2015). Clinicopathologic implications of eukaryotic initiation factor 3f and Her-2/neu expression in gastric cancer. Clin. Transl. Sci..

[16] Li G, Wang N, Sun C, Li B (2014). Decreased expression of eukaryotic initiation factor 3f is an adverse prognostic factor for stage I-III gastric cancer. World J. Surg. Oncol..

[17] Cheng Y, Jia C, Li G, Li H (2014). Expression of eukaryotic initiation factor 3f is associated with prognosis in gastric carcinomas. Oncol. Res. Treat..

[18] des Georges A (2015). Structure of mammalian eIF3 in the context of the 43S preinitiation complex. Nature.

[19] Wagner S, Herrmannová A, Šikrová D, Valášek LS (2016). Human eIF3b and eIF3a serve as the nucleation core for the assembly of eIF3 into two interconnected modules: the yeast-like core and the octamer. Nucleic Acids Res..

[20] Wang H (2013). Translation initiation factor eIF3b expression in human cancer and its role in tumor growth and lung colonization. Clin. Cancer Res..

[21] Wang Z, Chen J, Sun J, Cui Z, Wu H (2012). RNA interference-mediated silencing of eukaryotic translation initiation factor 3, subunit B (EIF3B) gene expression inhibits proliferation of colon cancer cells. World J. Surg. Oncol..

[22] Xu F (2016). Eukaryotic translation initiation factor 3B accelerates the progression of esophageal squamous cell carcinoma by activating β-catenin signaling pathway. Oncotarget.

[23] Choi YJ, Lee YS, Lee HW, Shim DM, Seo SW (2017). Silencing of translation initiation factor eIF3b promotes apoptosis in osteosarcoma cells. Bone Jt. Res..

[24] Zang Y (2017). Eukaryotic translation initiation factor 3b is both a promising prognostic biomarker and a potential therapeutic target for patients with clear cell renal cell carcinoma. J. Cancer.

[25] Lv H (2014). Epithelial cell-derived periostin functions as a tumor suppressor in gastric cancer through stabilizing p53 and E-cadherin proteins via the Rb/E2F1/p14ARF/Mdm2 signaling pathway. Cell Cycle.

[26] Molaei F, Forghanifard MM, Fahim Y, Abbaszadegan MR (2018). Molecular signaling in tumorigenesis of gastric cancer. Iran. Biomed. J..

[27] Kuai WX (2012). Interleukin-8 associates with adhesion, migration, invasion and chemosensitivity of human gastric cancer cells. World J. Gastroenterol..

[28] Ji W (2015). Role of p53β in the inhibition of proliferation of gastric cancer cells expressing wild-type or mutated p53. Mol. Med. Rep..

[29] Zhang GH (2015). Distinct novel quinazolinone exhibits selective inhibition in MGC-803 cancer cells by dictating mutant p53 function. Eur. J. Med. Chem..

[30] Zhang L (2015). Arsenic sulfide, the main component of realgar, a traditional Chinese medicine, induces apoptosis of gastric cancer cells in vitro and in vivo. Drug Des. Devel Ther..

[31] Wen F (2012). The tumor suppressive role of eIF3f and its function in translation inhibition and rRNA degradation. PLoS One..

[32] Doldan A (2008). Loss of the eukaryotic initiation factor 3f in pancreatic cancer. Mol. Carcinog..

[33] Eftang LL (2013). Up-regulation of CLDN1 in gastric cancer is correlated with reduced survival. BMC Cancer.

[34] Lopes AI, Vale FF, Oleastro M (2014). Helicobacter pylori infection—recent developments in diagnosis. World J. Gastroenterol..

[35] Zheng F (2017). Is it a protective factor of Helicobacter pylori infection in overall survival of all gastric cancer? evidence from meta-analysis. J. Environ. Pathol. Toxicol. Oncol..

[36] Fazeli Z, Alebouyeh M, Rezaei Tavirani M, Azimirad M, Yadegar A (2016). CagA induced interleukin-8 secretion in gastric epithelial cells. Gastroenterol. Hepatol. Bed Bench.

